# Evaluation of the elastic Young’s modulus and cytotoxicity variations in fibroblasts exposed to carbon-based nanomaterials

**DOI:** 10.1186/s12951-019-0460-8

**Published:** 2019-02-23

**Authors:** Homero F. Pastrana, Alexander X. Cartagena-Rivera, Arvind Raman, Alba Ávila

**Affiliations:** 1 Departamento de Ingeniería Eléctrica y Electrónica, Universidad de Los Andes, Bogotá D.C., Colombia; 2 Centro de Microelectrónica, Universidad de los Andes (CMUA), Bogotá D.C, Colombia; 3 School of Mechanical Engineering, Purdue University, West Lafayette, IN USA; 4 Birck Nanotechnology Center, Purdue University, West Lafayette, IN USA; 5 Present Address: Section on Auditory Mechanics, National Institute on Deafness and Other Communication Disorders (NIDCD), National Institutes of Health (NIH), Bethesda, MD USA

**Keywords:** Actin cytoskeleton, Carbon-based nanomaterials, Atomic force microscopy, Young’s modulus, Cell mechanics, Dimensionality, Cytotoxicity

## Abstract

**Background:**

The conventional approaches to assess the potential cytotoxic effects of nanomaterials (NMs) mainly rely on in vitro biochemical assays. These assays are strongly dependent on the properties of the nanomaterials, for example; specific surface area (SSA), size, surface defects, and surface charge, and the host response. The NMs properties can also interfere with the reagents of the biochemical and optical assays leading to skewed interpretations and ambiguous results related to the NMs toxicity. Here, we proposed a structured approach for cytotoxicity assessment complemented with cells’ mechanical responses represented as the variations of elastic Young’s modulus in conjunction with conventional biochemical tests. Monitoring the mechanical properties responses at various times allowed understanding the effects of NMs to the filamentous actin cytoskeleton. The elastic Young’s modulus was estimated from the force volume maps using an atomic force microscope (AFM).

**Results:**

Our results show a significant decrease on Young’s modulus, ~ 20%, in cells exposed to low concentrations of graphene flakes (GF), ~ 10% decrease for cells exposed to low concentrations of multiwalled carbon nanotubes (MWCNTs) than the control cells. These considerable changes were directly correlated to the disruption of the cytoskeleton actin fibers. The length of the actin fibers in cells exposed to GF was 50% shorter than the fibers of the cells exposed to MWCNT. Applying both conventional biochemical approach and cells mechanics, we were able to detect differences in the actin networks induced by MWCNT inside the cells and GF outside the cell’s membrane. These results contrast with the conventional live/dead assay where we obtained viabilities greater than 80% after 24 h; while the elasticity dramatically decreased suggesting a fast-metabolic stress generation.

**Conclusions:**

We confirmed the production of radical oxygen species (ROS) on cells exposed to CBNs, which is related to the disruption of the cytoskeleton. Altogether, the changes in mechanical properties and the length of F-actin fibers confirmed that disruption of the F-actin cytoskeleton is a major consequence of cellular toxicity. We evidenced the importance of not just nanomaterials properties but also the effect of the location to assess the cytotoxic effects of nanomaterials.

**Electronic supplementary material:**

The online version of this article (10.1186/s12951-019-0460-8) contains supplementary material, which is available to authorized users.

## Background

The incorporation of carbon-based nanomaterials in novel and everyday technologies requires to overcome its potential toxic effects by its beneficial properties; it is particularly true for those technologies in intimate contact with biological structures [1]. The assessment of CBN’s cytotoxicity has been highly debated recently. The reason is that the CBNs have several analytical obstacles, such as optical and chemical interference with current toxicity assay reagents, among them: adsorption of assay molecules by nanomaterials, increased reactivity of reagents, and shading effects of absorbance methods [2–5]. These interference behaviors raise concerns about the reliability of toxicity assays that may lead to uncertainty and contradictory results [6–9].

In light of these problems, international organizations such as the National Institute for Occupational Safety and Health (NIOSH), the Organization for Economic Cooperation and Development (OECD), the European Community (EC), and various non-governmental organizations (NGOs) are urging the development of label-free in vitro methods that avoid interference with NMs and improve assays’ reliability [10, 11]. New label-free approaches to identify NMs’ effects on biological structures include characterization of mechanical cell response due to deleterious changes in the cytoskeleton, impedance variations due to the loss of cells’ membrane integrity and proteomics to identify gene expressions under metabolic stress has been developed [12–16].

Our study is focused on the cellular mechanical response, which mainly depends on cytoskeletal integrity [17]. Cytoskeleton damage is a sign of cellular homeostasis dysfunction, due to cytotoxic products such as high concentration of radical oxygen species (ROS) [18]. Therefore, cytoskeleton’s structures disruptions can be associated with cells metabolic stress. The cytoskeleton integrity is shaped by three main structures: actin fibers, intermediate fibers, and microtubules. The concentrations of these structures change on different cells depending on their biological function. For example, the fibroblasts’ cytoskeleton is rich in actin fibers. The excess of ROS results in additional glutathione residues to the actin monomers during the polymerization process of F-actin [19]. Secondary to the glutathionylation, the F-actin cytoskeleton no longer possesses straight filaments across the cytoplasm, but instead an aggregation of actin monomers. The damage on actin fibers provides evidence of cellular stress due to ROS production. The lack of F-actin filaments results in lower Young’s modulus values [20–24]. Recently, excessive ROS production has been demonstrated to be exogenously induced by CBNs [25–29]. The increased production of ROS by CBNs can be linked to the biomechanical changes on fibroblast, specifically to lower Youngs modulus. However, the quantitative connections related to the ROS production and the cell mechanical properties with the CBNs’ dimensionality, such as the cylindrical MWCNT with one dimension out the nanoscale against planar GF with two dimensions, remains poorly understood. Molecular dynamics analysis, as well as experimental studies, had shown that 2D carbon nanomaterials have enhanced protein adsorption from culture media [30, 31]. This observation suggests that the GF depletes the protein availability for cell growth, faster than MWCNT; leading to metabolic stress and ROS production [32–35]. As a consequence, the cytoskeleton (F-actin network) will be disrupted, and the cell stiffness (Young’s modulus) decrease.

The ability to adsorb proteins by planar CBNs has been extensively reported [36]. When bacteria are exposed to GFs, there is dramatic adenine adsorption on the *E. coli* membrane by the GF surface destroying the bacteria inducing death [37]. Furthermore, MWCNT instead of modifying the protein adsorption, it had been shown to interact mechanically with actin cytoskeleton fibers possibly reinforcing its cellular structure resulting in a higher Young’s modulus [23].

Our work reveals a novel CBNs dimensionality relationship between the biomechanical responses of NIH3T3 fibroblast and CBNs’ toxicity. Strikingly, after cells exposed to carbon-based nanomaterials for only 2 h a considerable reduction in cellular mechanical properties is observed, whereas no significant production in ROS is measured. After 24 h, cells exposed to planar-shaped GFs produced twice as many ROS and exhibited a twofold decrease in Young’s modulus in contrast to cells exposed to cylindrical-shaped MWCNTs, even though that the specific surface area (SSA) of MWCNTs is double than the GFs SSA. Thus, we observed that the shape of CBN strongly affects the cellular cytotoxicity than their SSA. In both cases, no major variation on the cell viability was observed by biochemical methods (live/dead cell assays). To the best of our knowledge, this report is the first work to assess ROS production, cell’s mechanics and viability with CBNs dimensionality as a direct result of the disruption of actin stress fibers. The cytotoxicity assessment using cell mechanics adds a new dimension to the traditional biochemical assays and can be used to provide complementary information about biological interactions with nanomaterials.

## Results

### Characterization of carbon-based nanomaterials

Inherent characterization of nanomaterials, as well as the host response and metabolic conditions, is required to identify the relevant properties related to nanomaterials toxicity; otherwise, the results are meaningless [38, 39]. We focused the characterization of MWCNT and GF on the main physical–chemical properties related to cells’ toxicity: size/size distribution, shape, surface area, composition, impurities, and surface charge [40]. Table 1 summarizes the characterization results carried out in phosphate buffer solution (PBS) and culture media (DMEM) as well as the information provided by the manufacturer. Among the NMs properties, SSA has been widely accepted as the dominant toxicity predictor, since a greater SSA is associated with higher reactivity with cellular structures, in many cases due to a major ROS production [41]. However, other characteristics related to shape and dimensionality can be determinant on NMs behavior into the organisms. Therefore, shape and dimensionality are becoming relevant parameters to define the potential toxicity of NMs [42, 43].

**Table 1:** Physical and chemical CBNs characterization

Material	Shape	Size		SSA (m^2^ g^−1^)		Composition (%)	
		By manufacturer	By authors	By manufacturer	By authors	By manufacturer	By authors
*Graphene oxide flakes (GFs)* Cat. xGnP Grade H	1D flat material	Diameter: 3 μm Height: 15 nm	3 ± 1.2 μm 25 ± 15 nm	50–80	74.6 ± 1.8	C: 97.37 Cl: 0.2 Fe: 0.55 Ni: 1.86 S: 0.02	97.36 1.2 1.9 0 0
*MWCNT* Cat. US4311	2D fiber like material	Diameter: 35 nm Length: 1 μm	80 ± 7 nm 3 ± 1.5 μm	110	141.6 ± 1.2	C: 96.87 O: 3.13 Ni: 0	96.18 0 1.11

Our results of physicochemical characterization (size, SSA, and impurities) of the CBNs differ from the ones provided by manufacturers. Standardizations labs have noticed these discrepancies between the characterization from the manufacturer and final user as significant constraints on nanomaterials’ toxicological studies [38]. The shape and size of both nanomaterials were measured using TEM. The GFs exhibited a planar shape up to 3 ± 1.2 μm in length and 25 ± 15 nm in height. The MWCNTs exhibited a cylindrical shape with diameters 80 ± 7 nm and a length of 3 ± 1.5 μm; see Additional file 1. The SSA of the GFs was 74.6 ± 1.8 m^2^ g^−1^ approximately half that of the MWCNTs 141.6 ± 1.2 m^2^ g^−1^. Elemental analysis revealed that both CBNs contained impurities, in the case of GF chlorine 1.2% and iron 1.9% and the case of the MWCNT iron was not detected either reported by the manufacturer only nickel at 1.11% was detected by the authors. Impurities in CBNs are related with the catalysis of the Fenton reaction, especially iron impurities, which stimulates ROS production in cells, however, the concentrations presented in the GF are not likely to induce severe damage on mammalian cells [44–46]. It has been previously demonstrated that rather than introduce toxicity by the presence of iron, MWCNTs promotes iron metabolism sequestration inducing cellular inflammation [45, 47]. Due to the greater SSA and the facilitated cellular uptake of the MWCNT we expected significant lower viability on cells exposed to MWCNTs than cells exposed to GFs [48]. Here we evidenced how the extracellular CBNs are as important as intracellular to determine the cells toxicity induced by these materials and its dependence on CBNs shape and dimensionality.

### Cellular CBN uptake

**Fig. 1:**
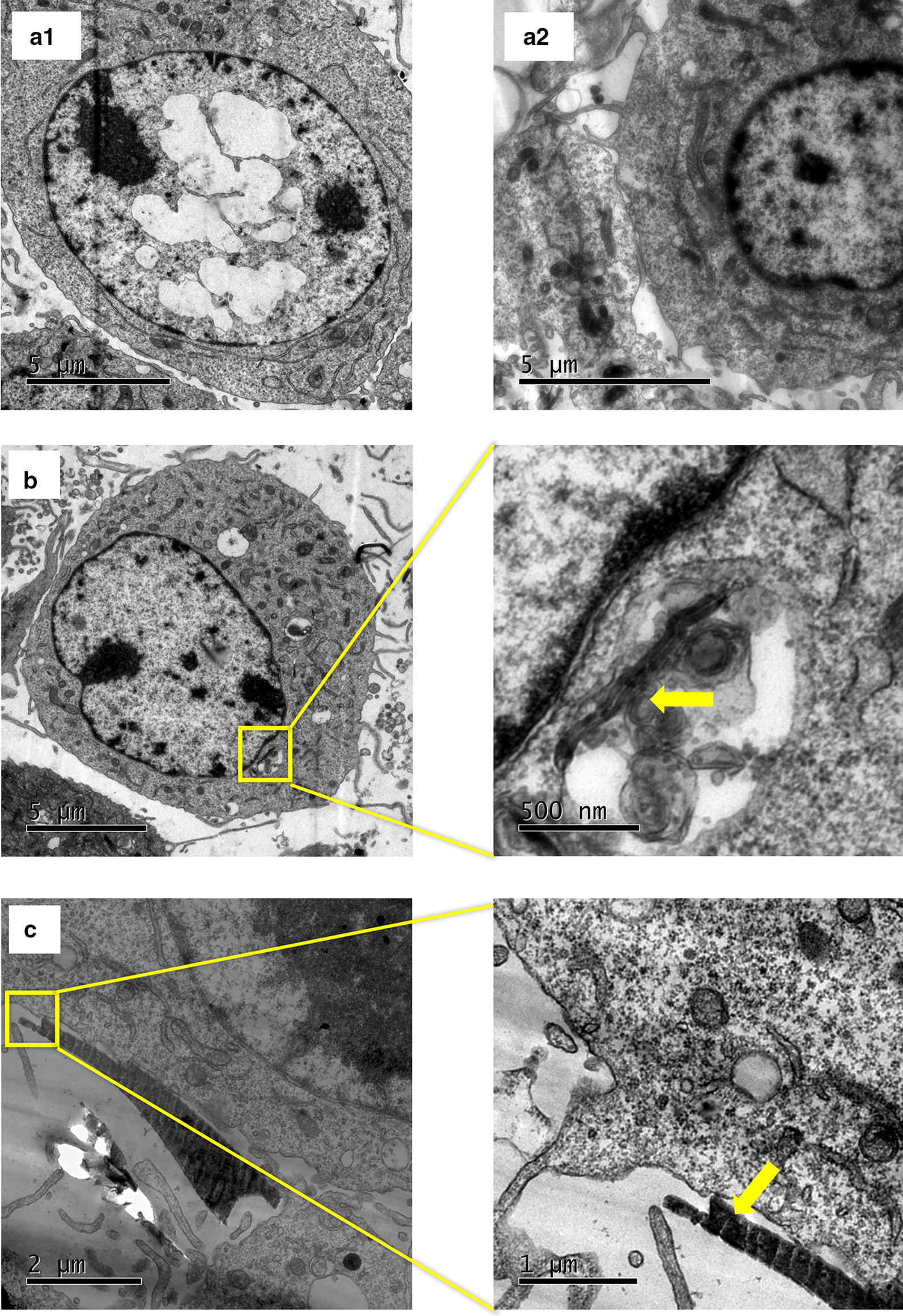
CBN uptake by NIH3T3. **a1** and **a2** control cells, no exposure to CBNs. **b** Cells exposed for 24 h to MWCNTs. The yellow arrow highlights an MWCNT into a vacuole near the nucleus. **c** GFs outside the cell near the cell’s membrane. The yellow arrow indicates a GF

The effective uptake of CBNs into the cells depends on nanomaterial properties (size, shape, surface charge, protein corona) and the cell line (fibroblast, macrophage, neuron) [49]. We selected NIH3T3 fibroblasts since phagocytosis is not an active process in these cells [50]. Therefore, the cellular uptake will depend more on the nanomaterials properties rather than the cellular activity of engulfing foreign bodies like macrophages [51]. The cells were exposed to 50 µg/mL of CBNs for 24 h. Then were fixed and prepared for TEM imaging. Figure 1 shows the TEM images of NIH3T3; the control cells are in row A, the cell is characterized by a large nucleus with no significant changes in cytoplasmic organelles. The cells exposed to MWCNT shows almost two times the number of mitochondria and some vesicles with MWCNT inside, Fig. 1b. Cells exposed to GFs did not show NMs in their cytoplasm, Fig. 1c; the GF remained outside the cells and appeared in some cases to adhere to the cellular membrane. This phenomenon is relevant to understand how the intra and extracellular effects on cells toxicity from both CBNs are triggered. The planar sheet shape and larger size of the GFs than the MWCNTs are likely responsible for modifications to the cells’ environment, such as media protein adsorption [30]. GF exhibits an enhanced protein sequestration ability compared with MWCNTs. However, MWCNTs seems to exert more intracellular effects interfering with cellular structures and breaking lysosomal vesicles increasing the ROS production [26].

### Cell viability

**Fig. 2:**
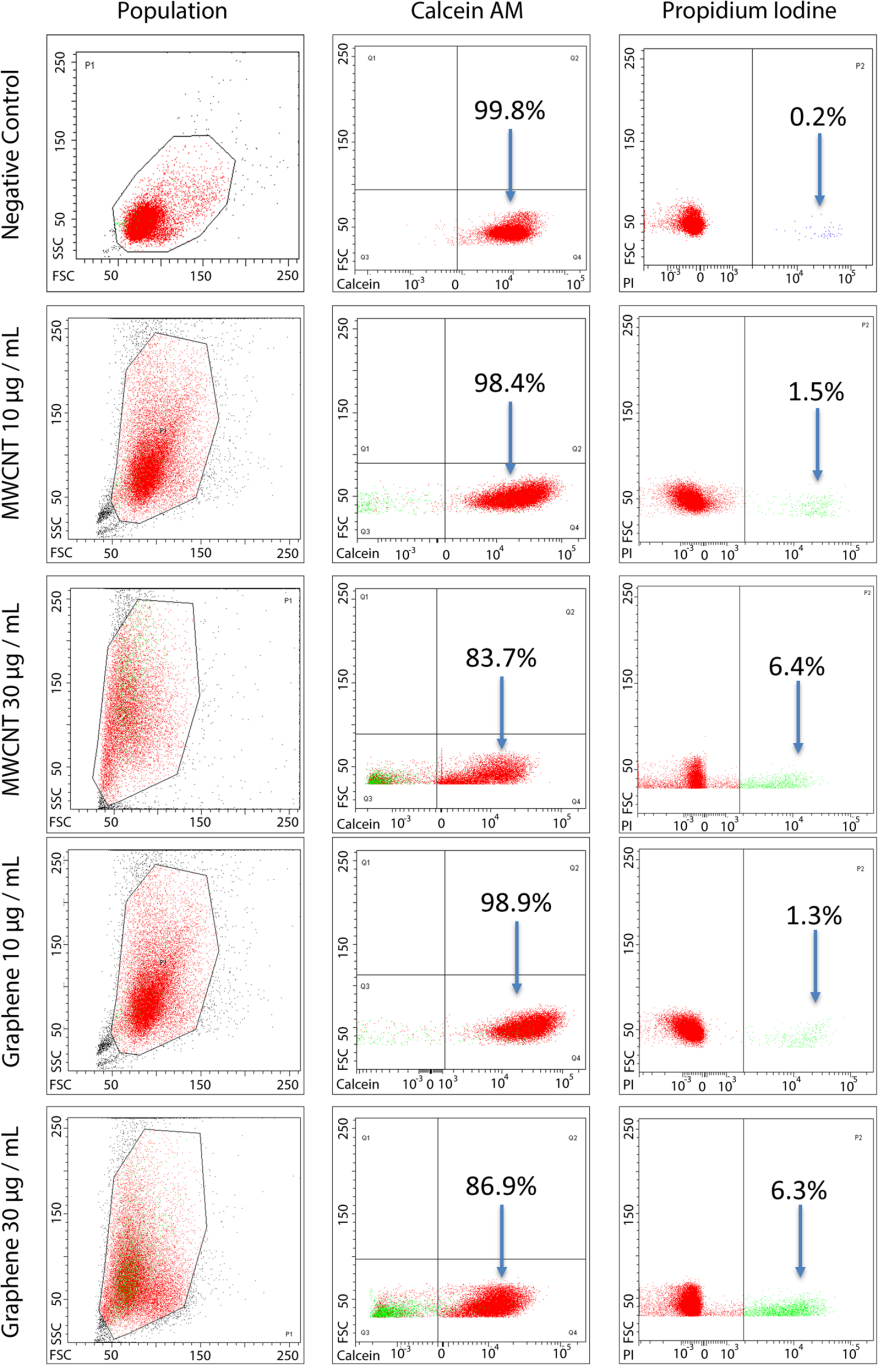
Flow cytometer results of the live/dead assay. NIH3T3 fibroblasts were exposed for 24 h to 10 and 30 µg/mL CBNs. The population column refers to the gated group of cells measured to identify calcein (live) and PI (dead) populations. A concentration-dependent decrease of cell viability is observed. No significant differences were observed between the two nanomaterials by *t* test

The conventional biochemical live/dead assay is frequently used to report the cellular esterase activity and the membrane integrity as determinants of cell viability affected by direct exposure to CBNs. At low concentrations, 10 µg/mL, cells’ viability had a marginal reduction of 2% for both CBNs. Higher concentration of 30 µg/mL shows cells viability decrease; in the case of MWCNT by 16.3% and the case of GF by 13.1%. We also exposed the cells to a concentration of 50 µg/mL, however, at this concentration a large number of detritus did not allow a reliable reading by the flow cytometer. Figure 2 presents the dispersion diagrams of the populations expressing calcein-AM and propidium iodine in the live/dead assays. The results from live/dead assay confirms a slightly higher cytotoxic effect expected from MWCNT probably secondary to a larger SSA, nickel impurities, and facilitated cell uptake.

### ROS generation

**Fig. 3:**
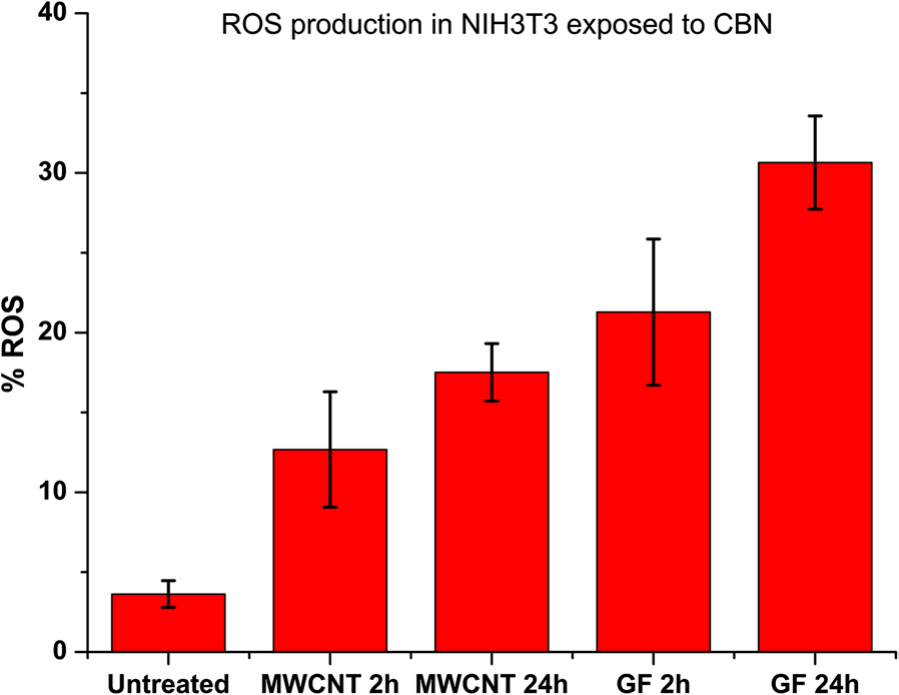
Time-dependent ROS production on untreated and treated NIH3T3 fibroblasts with carbon-based nanomaterials (MWCNTs and GFs) after 2 h and 24 h. GFs induced greater production of ROS in cells than MWCNTs. Significant at *p* < 0.05, n: 1000 points per group, t-test

The ROS production was measured by a laser-enabled analysis and processing system (LEAP) using dihydroethidium (DHE) as a marker. In the presence of oxygen radicals, the molecules of 2-hydroxyethidium intercalate with the DNA and fluoresce red (576 nm) [52]. The cells exposed to both CBNs, at a concentration of 50 µg/mL, show an increased ROS production. The percentage of cells expressing ROS production was two times higher on cells exposed to GF than in those exposed to MWCNT, Fig. 3. The GFs in the culture media was able to increase the number of cells expressing ROS production up to 21% as quickly as 2 h after exposure, and 32% after 24 h resulting in a greater deleterious effect on cellular metabolism. The ROS production results differ from the viability assay, reported above, where cells treated with GF had slightly lower toxicity compared with the cells exposed to MWCNT. Cells labeled with DHE indicates metabolic stress by ROS production that does not mean imminent death [53]. Therefore, it is possible to have stressed cell also labeled as viable by calcein marker. To the best of our knowledge, this work represents the first demonstration of such rapid cellular toxicity that may be critical to early diagnostics of potential toxic effects.

### F-actin cytoskeleton disruption

The length of actin stress fibers measured using Alexa Fluor 488 conjugated-phalloidin marker evidenced significant shortening on cell exposed to both CBNs. The length of the actin fibers can be used as a descriptor for the cytoskeleton disruption that explains the changes on cells Young’s modulus induced by the exposure of CBNs, at a concentration of 50 µg/mL.

**Fig. 4:**
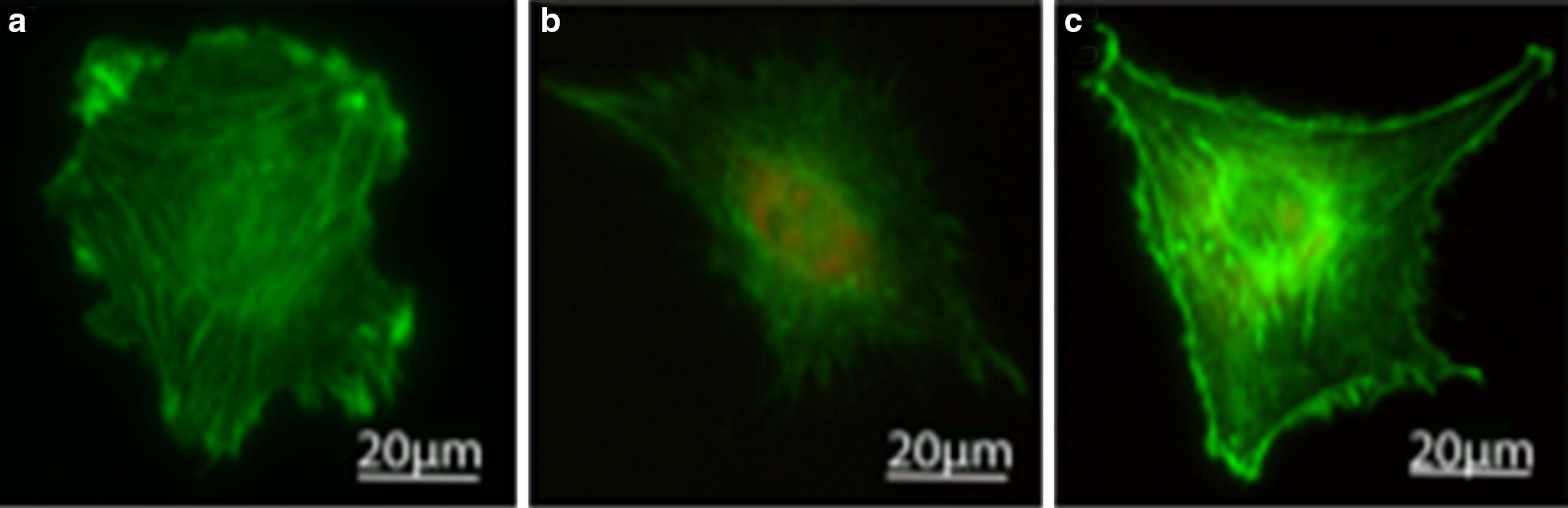
Fluorescent staining of NIH3T3’s cytoskeleton: **a** control cell; F-actin (green) is highly organized with stress fibers extending continuously along the cells. **b** The cell exposed to GF for 24 h; the F-actin integrity is compromised and is only observed somewhat organized close to the nucleus. **c** The cell exposed to MWCNTs for 24 h; the F-actin is dispersed around the cytoplasm. The control cells exceeded, up to eight times, the length of the fibers of the cells exposed to both CBNs (MWCNT and GF). The fiber length was measured by image processing on ImageJ skeleton algorithm. Red indicates the DHE dye, showing the levels of ROS within cells exposed to CNBs

In particular, shorter stress fibers are associated with reduced cell adhesion and motility [54], which are critical cellular processes for cell division and migration. Figure 4 shows the actin fibers (in green), the actin stress fibers in untreated cells are continuous lines across the cell’s cytoplasm Fig. 4a, with an average length of 13.7 ± 2.3 µm. By contrast, actin stress fibers of cells exposed to GF were eight times shorter (1.7 ± 0.7 µm) (Fig. 4b). The cells exposed to MWCNT had their fibers six times shorter (2.1 ± 1 µm) (Fig. 4c). Disaggregation of actin filaments has resulted in reducing the cell’s Young’s modulus, and it has been used as a correlation between the F-actin network and the cell’s mechanical stability [55].

### Young’s modulus maps

**Fig. 5:**
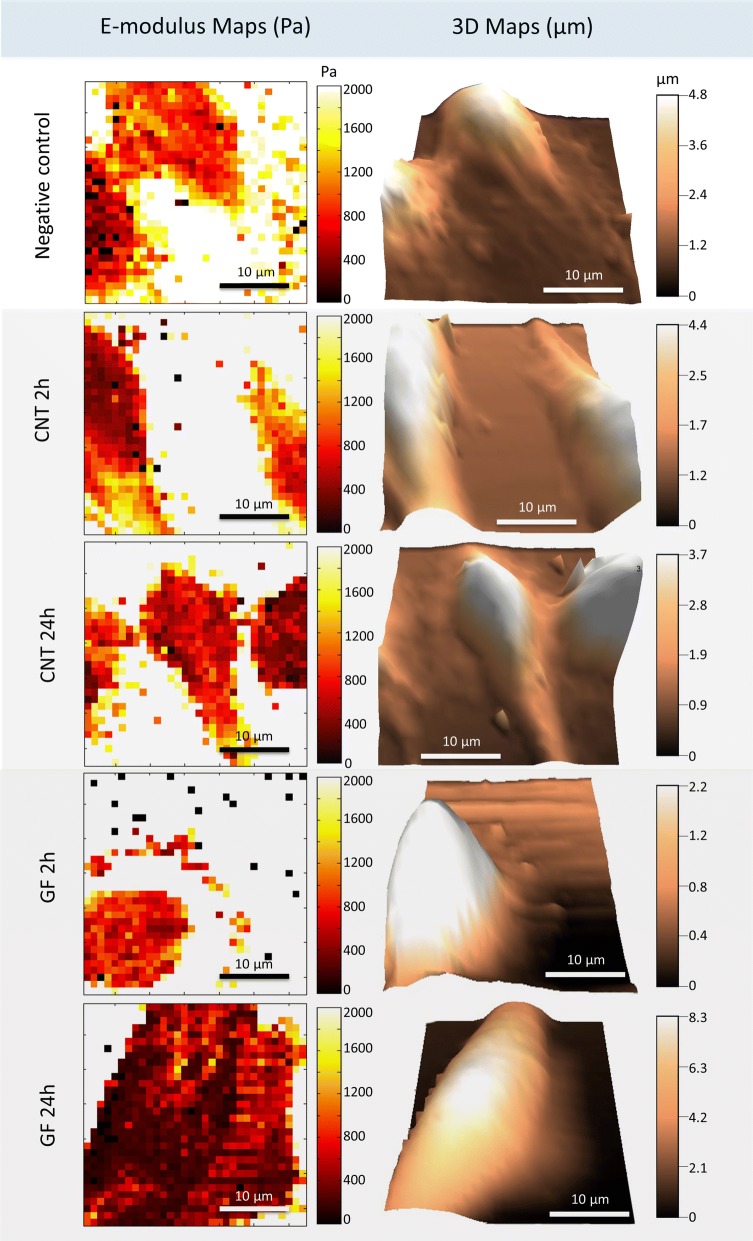
Elastic Young’ modulus maps and three-dimensional topographic reconstruction of live NIH3T3 in culture media before and after exposure to CBNs: **a** unexposed cells, **b** cells exposed 2 h to MWCNTs, **c** cells exposed 24 h to MWCNTs, **d** cells exposed 2 h to GFs and **e** cells exposed 24 h to GFs

Atomic force microscopy is a powerful tool for the measurement of mechanical properties of cells in their physiological environment [56–60]. Details of the operating principle and applications of AFM are well documented in the literature [61, 62]. We used the standard force–volume AFM mode to generate topographic, and elasticity map sought to determine the cell’s stiffness (Young’s modulus) of untreated and CBN-treated (MWCNT and GF) fibroblasts after 2 and 24 h of exposure, to 50 µg/mL concentration, Fig. 5.

**Fig. 6:**
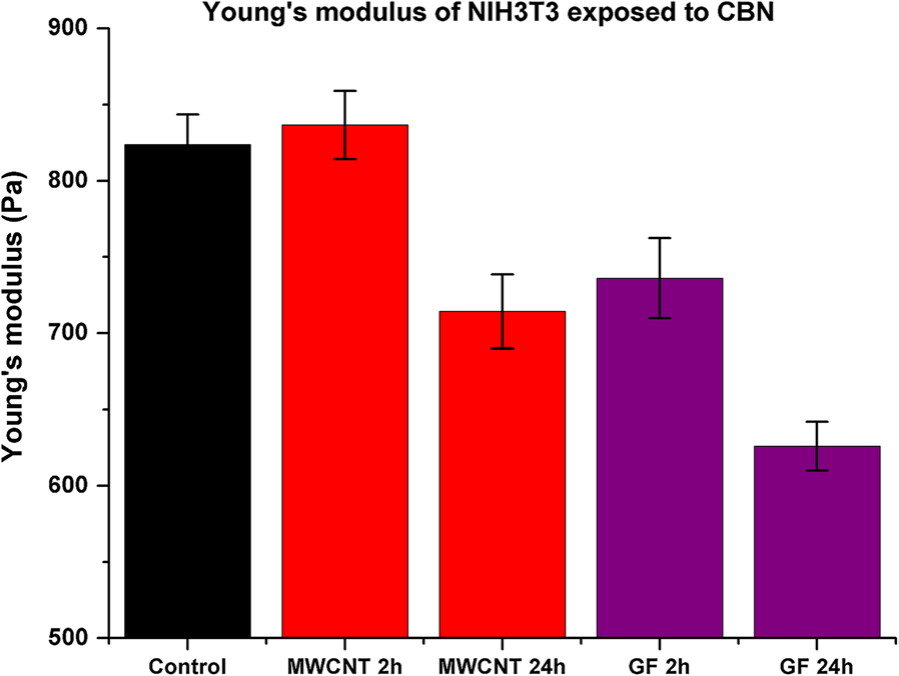
Young’s modulus differences between untreated cells and cells exposed to MWCNTs and GFs. The elastic Young’s modulus of fibroblast cells after exposure to GFs significantly decreases by ~ 20% than the control cells, whereas cells exposed to MWCNTs decreased ~ 10%. The force/volume measurements were done at least 22 points per cell. Each group of measurements includes 13 cells. Significant at *p* < 0.05 by t-test

Figure 5 shows the Young’s modulus maps and the 3D reconstruction of the cells’ topography. The darkest areas in the Young’s modulus maps represent the lowest modulus; the cells exposed to MWCNT and GF after 2 h show lighter areas compared with the maps after 24 h. Figure 6 presents the quantified data from all the cells and measured points (1437 points in 64 cells). The Young’s modulus of the cells exposed to MWCNT after 2 h had a slight increase, this effect has been reported by different authors after the administration of SWCNT, mentioning that it can be related to the reinforcement by nanomaterials to the cytoskeleton fibers (actin stress fibers) [63, 64]. Nevertheless, after 24 h of exposure to MWCNT the Young’s modulus dramatically decrease by 13.6% compared with 2 h results. In the case of GF, no previous studies were found analyzing the mechanical response of cells after the exposure to them.

Our results evidenced an initial decrease of 8.6% after 2 h of exposure contrary to the initial increase observed on cells exposed to MWCNT after 2 h. The decrease of the Young’s modulus, 24.6% lower, is more evident after 24 h. The metabolic impairment of the cells due to the ROS production and cytoskeleton fiber shortening is correlated with the mechanical response of the cells. On the other hand, the live/dead assay showed non-significant variations on the viability of cells exposed to MWCNTs and GFs; despite the lower concentration of CBNs tested. This incongruency suggests the need for alternative techniques, to validate the results by a different mechanism not affected by biochemical interactions, such as cellular stiffness.

### Protein adsorption by CBNs

**Fig. 7:**
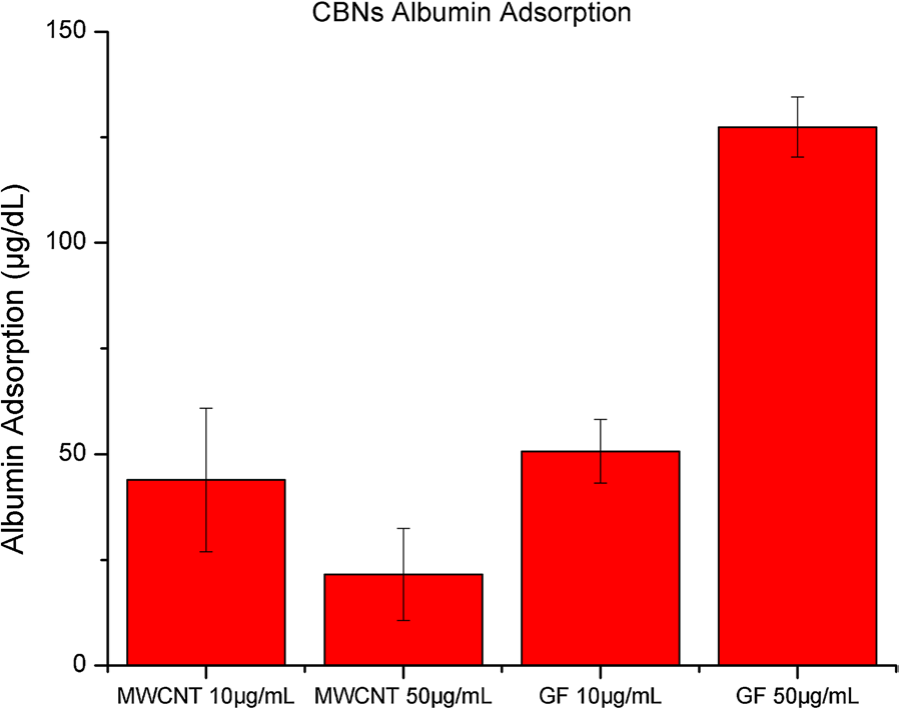
Protein concentration in the supernatant after exposure to CBNs for 24 h at a concentration of 10 µg/mL and 50 µg/mL

CBNs adsorption of protein is linked to their dimensionality. It is reported that 2D nanomaterials as GF are more likely to provide a better surface for protein residues anchorage where the proteins do not require as much deformation as with more rigid 1D CNTs. Therefore, it is a shape dependence of the ability of CBNs to adsorb media proteins. We comparatively evaluated the albumin adsorption between GF and MWCNT to determine the potential extracellular effect of both nanomaterials in cells metabolism due to protein sequestration. We used the bicinchoninic acid assay (BCA) to quantify the concentration of the BSA in the supernatant after the administration of CBNs. We used two concentrations 10 and 50 µg/mL in DMEM enriched with 10% of BSA. We found that both CBNs reduced the concentration of media proteins. After 24 h of exposure to MWCNTs adsorbed 21.5 µg/mL of albumin, in contrast, the exposure to GFs had albumin adsorption of 127 µg/mL, see Fig. 7. It demonstrates the higher capability for planar CBNs to sequester proteins compared to cylindrical shaped CBNs. It is more evident when the adsorbed proteins are calculated considering the SSA; in the case of MWCNT, its protein adsorption regarding the SSA is 3.04 µg/m^2^ while the GF had protein adsorption of 33.5 µg/m^2^. There is more protein adsorption, ten times, in GF regarding their SSA. This behavior has been previously demonstrated via molecular dynamics simulations, where π–π stacking with the aromatic residues of proteins showed to be reduced in cylindrical nanostructures, limiting the adsorption [30, 31, 36, 65]. The lower availability of media protein can be considered as a major cause of cells toxicity induced by GF on NIH3T3 fibroblasts since the presence of these nanomaterials into the cells were not proved by TEM images.

## Discussion

Our results indicate that NIH3T3 fibroblasts exposed to MWNT and GF nanomaterials are under metabolic stress, which activates mechanisms such as ROS production, and mechanical regulation to maintain their homeostasis. We also found that F-actin cytoskeleton integrity was disrupted not only by the administration of CBNs in the cytoplasm but also by the overproduction of ROS in the cells and the protein depletion in culture media. These three pathways for cell toxicity altered the cells Young’s modulus in time and NM dependent manner. After 2 h of exposure, fibroblasts exposed to GFs exhibited a fast-mechanical regulation, a 10% decrease in modulus and after 24 h, the modulus had decreased by 20%. By contrast, fibroblasts exposed to MWCNTs exhibited 4% increase in Young’s modulus after 2 h and a dramatic decrease of 10% after 24 h of exposure.

These results regarding the relationship of the Young’s modulus of cells to the nanomaterials’ dimensionality are a novel approach to understand the cytotoxic effects. Liu et al., presented an innovative approach from the single-cells assessment of mechanical response by compressing using a single point measurement of the Young’s modulus of the macrophages after exposure of silver nanoparticles. They found a correlation between the filamentous actin cytoskeleton disruption and the cells’ Young’s modulus decrease on cells that had phagocytose the nanoparticles [58, 59]. However, no analysis of cells stiffness at multiple points of the cells were done to map the cell localized heterogeneity, as wells as the assessment of the extracellular effects and dimensionality of the nanomaterials were done.

Previous works have concentrated on studying the toxicity effects based on the SSA [41]. The SSA is a critical parameter in in vitro assays that evaluate the response of cells to nanomaterial exposure, as detected on the basis of catalytic activity, acidity/alkalinity, absorption, fluorescence signaling, adsorption capacity, and metabolic dynamics [3]. According to previous studies, a larger SSA may lead to an increase in toxicity, as evaluated by traditional in vitro assays focused on the previously listed responses [48]. Therefore, we would expect higher toxicity from MWCNTs than from GFs. However, our cytotoxicity study, which combines biochemical assays with evaluations of the response of mechanical cell properties to exposure to CBNs, demonstrates that, in the case of GFs, a smaller SSA results in a substantial change in elasticity and ROS production. Murray et al. [66], evidenced that SSA no necessary predict the toxicity response of CBNs. Zimmer et al. [59], study the effect SiO_2_ NP on the cell’s membrane stiffness, cells exposed to NPs evidenced a concentration-dependent decrease of cell membrane modulus.

Our study shows the dependence of the Young’s modulus on exposure time. Also, we evidenced that after 2 h of exposure to GFs the cells’ volume decrease. It is probably related to the water displacement outside the cells by osmotic pressure due to the high affinity of proteins such as albumin to be adsorbed by GFs increasing the oncotic pressure. By contrast, at longer exposure times (24 h), the cells’ metabolic stress was induced by ROS production, and cytoskeleton damage, which led to an increase in cell volume, probably as result of sodium/potassium pumps dysfunction [67].

We propose that cell toxicity is affected by CBNs’ shape, and not only by their SSA. The shape is critical for protein absorption, which may be enhanced on planar nanomaterials such as graphene flakes. Our experimental protein absorption results demonstrated higher adsorption rates for GFs than for MWCNTs, which led to a significant decrease of the availability of proteins for cell homoeostasis, increasing the ROS production after 24 h of exposure to 32% for cells exposed to GF and 17% for cells exposed to MWCNTs. These results suggest that the CBNs’ shape affects cell homeostasis. Other reports have indicated that the similarity in shape and dimensions of MWCNTs with the actin stress fibers, once in the cell, could emulate the fibers, resulting in reinforcement of the cytoskeleton [31]; this effect could explain the results observed for NIH3T3 fibroblasts exposed to MWCNTs for 2 h. By contrast, a planar shape, such as that of the GFs, results in cellular membrane deformation associated with enhanced protein adsorption, a lower Young’s modulus and higher ROS production compared to those associated with exposure to MWCNTs, despite their lower SSA.

We emphasize that the in vitro toxicity assessment is improved by considering the measurement of mechanical properties in living cells exposed to carbon-based nanomaterials, here demonstrated to overcome the interference of biochemical reagents of the current cytotoxicity kits. New indicators, such as the one described herein, can provide insights into the routes of NMS within cells and the dominant mechanism of their interactions.

## Conclusions

We established a correlation between the CBN toxicity and cells’ physical parameter (shape and elasticity) that has not been previously identified by the conventional live/dead viability assay. The results demonstrated that MWCNTs and GF induced cytotoxicity in fibroblast at different timescales. The F-actin measurement indicated a time depending disruption as well as dimensionality relation. Cells exposed to GFs resulted in shorter actin fibers after 24 h than the fibers in cells exposed to MWCNT. We found that the greater SSA of MWCNT were not a good predictor of cells toxicity by CBNs as their shape and dimensionality. We found a drastic reduction of 20% in the Young’s modulus of cells exposed to GF compared with the 10% of those exposed to MWCNT.

The modulus variations correlated with the generation of ROS and the viability assays demonstrated the weak reliability of the evaluation of cytotoxicity based only on one endpoint with traditional biochemical assays: live/dead. In the experiments, the viability assays lead to infer that both CBNs are innocuous. However, the introduced analysis of the length modifications in the actin networks once exposed to CBNs and their estimation of cell’s Young modulus indicates a deleterious effect on the mechanical stability of living cells that was opposite to the results of the viability assays. We understand the techniques are applied to different scales, viability conclusions came from an average of the cell culture, and Young’s modulus estimations from a single cell but the number of measurements 22 per cell and 13 cells per culture provides support to our findings and invite to think on the need of emerging toxicity assessment at the single level. In addition, the introduced force/volume technique permit to simultaneously quantify a single cells volume variation at different periods that has provided information of the process of cell death.

In summary, our results suggest that toxicity assessments need to consider additional techniques apart from the traditional, here demonstrated with the integration of the single the cells’ mechanical properties. Especially, techniques that will monitor changes measuring intracellular properties that are needed to understand NMs fate into the cells, the interaction with the cytoskeleton components and other organelles.

## Materials and methods

### Nanomaterials

#### Multiwall carbon nanotubes

Multiwall carbon nanotubes (US Research Inc, Houston, TX, USA) were suspended in 1× phosphate buffered saline (PBS) (Gibco, Waltham, Massachusetts, USA) at 1 mg/mL. One hundred microliters of the MWCNT suspension was added to 2 mL of DMEM (Gibco, Waltham, Massachusetts, USA) to obtain a final concentration of 50 µg/mL. Samples were not sonicated to simulate a direct biological contact under normal NMs exposure in the environment.

#### Graphene oxide flakes

Graphene oxide flakes (XG Sciences Inc, Lansing, MI, USA) were suspended in 1× PBS at 1 mg/mL. 100 µL of GF suspension was added to 2 mL of DMEM to obtain a final concentration of 50 µg/mL. Samples were not sonicated as well as MWCNT.

### Nanomaterials characterization

#### Transmission electron microscopy

The structural characteristics—size and shape—of both CBNs (MWCNTs and GFs) were analyzed using a JEOL JEM 1400 plus transmission electron microscope (Jeol, Tokyo, Japan; located at Fundación Santa Fe). Each sample, 10 µL at 1 mg/mL in 1× PBS, were placed on agar grids and dried. The PBS has removed washing with distilled water twice. The TEM images of the nanomaterials were carried out only in PBS. The size estimation of CBNs from TEM is the final result from averaging over 10 CBNs over 10 images.

#### BET surface area analysis method

The BET technique was used to measure the specific surface area of both CBNs (MWCNTs and GFs) using a Gemini VII 2360 (Micromeritics, Georgia, USA; located at Purdue University). The samples were dried for 6 h and loaded into the instrument; nitrogen was used as the adsorbate. The CBNs were not exposed to culture media prior to the BET characterization.

### Raman spectroscopy

Raman spectroscopy was used to structurally characterize both nanomaterials (MWCNTs and GFs) in powder form. 10 µg of each sample was placed on an XploRA confocal Raman spectrometer (Horiba Scientific, Kyoto, Japan; located at Universidad de Los Andes). The Raman spectrum was collected between 101 and 2801 cm^−1^.

### Cell culture

NIH3T3 (ATCC CRL-1213; American Type Culture Collection, Manassas, VA, USA) were selected to assess the toxic effects of CBNs since the fibroblast are responsible for extracellular matrix production and these cells are present during the injury/repair processes. We cultured the NIH3T3 in DMEM (Gibco, Waltham, Massachusetts, USA) containing a low concentration of d-glucose (1000 mg L^−1^), 10% fetal bovine serum (FBS) (Invitrogen, Carlsbad, CA), 1% penicillin–streptomycin (Invitrogen, Carlsbad, CA), and 0.1% amphotericin B (Sigma-Aldrich, St. Louis, MO). Cells were then seeded on a culture dish (Fluorodish, 50 mm, WPI, Sarasota, FL, USA) pre-coated with 0.1% gelatine in water (STEMCELL Technologies, Vancouver, British Columbia, Canada) at a concentration of 1.2 × 10^5^ cells/mL. The seeded cells were grown in the dish for 24 h before experiments and stored in an incubator at 37 °C under a 5% CO_2_ atmosphere to ensure complete spreading.

### Laser-enabled analysis and processing (LEAP)

NIH3T3 cells were seeded overnight in a specific 96-well plate (Cyntellect, San Diego, CA, USA) for laser-enabled analysis and processing (LEAP™) (Cyntellect, San Diego, CA, USA; located at Bindley, Purdue University) at 5 × 10^5^ cell/mL concentration in culture media. 15 µL of MWCNTs or GFs at 1 mg/mL was added to each well to a final concentration of 50 µg/mL. Then, cells were incubated for 2 and 24 h at 37 °C under 5% CO_2_ atmosphere. The medium was then removed, and the wells were washed thoroughly with PBS supplemented with Mg^2+^. Finally, calcein-AM green dye (Life Technologies, Carlsbad, CA, USA) for cell viability, DHE dye (Life Technologies, Carlsbad, CA, USA) for ROS production, and Hoechst 33342 dye (Life Technologies, Carlsbad, CA, USA) for DNA staining were added. Cells were stained for 30 min in the dark and at room temperature before being processed using LEAP. Three replicates per CBN were tested, each group has a complete field image of the well, see Additional file 1. The percentage of ROS production was calculated from the cells expressing DHE over the total of cells labeled with calcein.

### Flow cytometry

Cells were seeded in culture media overnight on 5 cm diameter Petri dishes at a concentration of 2.5 × 10^6^ cell/mL. After 24 h, 200 µL of MWCNTs or GFs at 1 mg/mL were added to each well to a final concentration of 10 and 30 µg/mL. Then, cells were incubated for an additional 24 h at 37 °C in an atmosphere with 5% CO_2_ and a relative humidity of 95%. The culture medium was then removed, and the wells were washed thoroughly with PBS supplemented with Mg^2+^. Later, cells were harvested using trypsin and centrifuged at 200×*g*. Subsequently, calcein-AM green dye (Life Technologies, Carlsbad, CA, USA) and propidium iodine (PI) (Life Technologies, Carlsbad, CA, USA) were added to stain the cells for 30 min in the dark at room temperature. Finally, the stained cells were measured using a flow cytometer (FACS Canto II, BD Bioscience, San Jose, CA, USA; located at Universidad de Los Andes), which counted to 10,000 events for 30 s. Each group was seeded and tested three times, see Additional file 1.

### TEM

NIH3T3 cells were seeded overnight in 3.5 cm diameter Petri dishes at 2.5 × 10^6^ cell/mL in culture medium. After 24 h, 100 µL of MWCNTs or GFs at 1 mg/mL were added to each well to a final concentration of 50 µg/mL. The cells were then incubated for 24 h at 37 °C under a 5% CO_2_ atmosphere. The medium was then removed, and the wells were washed twice with PBS 1× containing Mg^2+^. Next, cells were trypsinized, harvested, centrifuged, and the supernatant was removed. Cells were fixed with 3% formaldehyde for 30 min at room temperature. Subsequently, cells were treated in osmium tetroxide (Sigma-Aldrich, Cat 75632, CA, USA) and dehydrated in a graded series of ethanol. After dehydration, samples were embedded in LR White resin (Cat. L9774, Sigma-Aldrich, USA). Finally, samples were cut using a microtome (ThermoFisher, MD, USA) and loaded into a JEOL 1400 Plus TEM (Jeol, Tokyo, Japan; located at Fundación Santa Fe, Colombia).

### Immunofluorescence imaging

After incubating for 24 h, the cells were exposed to 50 µg/mL of CBNs (MWCNTs or GFs) in a fluorodish; the media was removed, and the cells were washed twice using PBS with Mg^2+^. Subsequently, DHE dye was added to the cells. Next, the cells were fixed using 3.7% formaldehyde for 10 min at room temperature and permeabilized in 0.2% Triton X-100 for 2 min. Then, Alexa Fluor 488 conjugated phalloidin (Cat A12379, Thermofisher, MD, USA) was added to label the actin cytoskeleton; the phalloidin toxin has a high affinity for F-actin subunits, stopping the normal denaturalization; thus, the actin fiber can be visualized when the phalloidin is labeled with a fluorescence compound. The cells were incubated at room temperature for 30 min to be visualized in an inverted epifluorescence Olympus IX71 microscope (Olympus, Tokyo, Japan). We measured the actin stress fiber length using the ImageJ software (National Institutes of Health, Bethesda, MD, USA) by segmenting the continuous line in the actin image representing stress fibers.

### AFM sample preparation

After 24 h of cell culture, CBNs (MWCNTs or GFs) were added to the medium at a final concentration of 50 g/mL. Cells were incubated for an additional 2 h and 24 h. The cells were then washed twice with warm culture medium (37 °C), and 2 mL of fresh medium was added to each dish. Cells were maintained at 37 °C during the AFM experiments using a Petri dish holder and a heater on the MFP-3D scanner X–Y stage (Asylum Research, Santa Barbara, CA, USA).

### Atomic force microscopy

The MFP3D-Bio AFM system (Asylum Research, Santa Barbara, CA, USA) located in the Birck Nanotechnology Center at Purdue University was used in this work. We used TR400PB soft microcantilevers (Olympus, Tokyo, Japan) with a nominal spring constant of 0.09 N/m, modified by attachment of a 5 µm borosilicate glass microsphere close to the tip (Novascan, Ames, IA, USA). The AFM probes were calibrated by constructing a force–distance (*F*–*Z*) curve on a mica substrate. The photodetector optical sensitivity was obtained by extracting the slope of the *F*–*Z* curve. The cantilever spring constant was calculated using the thermal fluctuations noise method [68]. The estimated microcantilever spring constant was 0.05–0.1 N/m. All the AFM imaging and measurements were performed under physiological conditions using culture media at 37 °C.

To estimate the elastic Young’s modulus of the fibroblast cells, the force–volume (*F*–*V*) mode was used. This AFM mode is based on the acquisition of low-speed, quasi-static *F*–*Z* curves predefined by the user grid of points, resulting in the simultaneous acquisition of topography and material properties maps [69]. In this study, the images were obtained with a resolution of 32 × 32 pixels, with an approximate acquisition time of 35 min per image. The relative trigger force used was 2 nN to minimize the cell deformation and the damage to the cells. The *F*–*Z* curve rate was set to 1 Hz (1 s) to minimize viscoelastic contributions [60]. We extracted the cell elasticity offline for each recorded *F*–*Z* curve by initially converting to force–indentation (*F*–*δ*) and subsequently fitting with the Hertz contact mechanics model [70]. The Hertz contact mechanics model for a rigid sphere indenting an infinite isotropic elastic half-space is

1 $$F_{ts} = \frac{4}{3}\frac{E}{{1 - \nu^{2} }}\sqrt R \delta^{{3/2}}$$

where *F*_*ts*_ is the tip-sample indentation force (N), *E* is the elastic Young’s modulus (Pa) of the sample, *δ* is the sample mean indentation, $\nu$ is the Poisson’s ratio (defined as 0.5 for soft live cells), and *R* is the microsphere radius. An important assumption in the analysis using this model is that the microsphere indentation is much smaller than the finite cell height (10–20% of the cell thickness) to minimize artifacts in extracted elasticity maps originating from contributions of the hard substrate due to the finite cell thickness [71]. To satisfy this assumption, data were analyzed up to indentations of 20%-pixel cell height.

### Protein quantification

The protein quantification was carried out using the Pierce bicinchoninic acid assay (BCA) (ThermoFisher, Cat 23225, MD, USA). To a 100-μL aqueous sample containing 10–100 μg protein, 2 mL of solution working reagent was added and incubated at 37 °C for 30 min. Then, after the sample had cooled to room temperature, the absorbance at 562 nm was measured. A calibration curve was constructed using dilutions of a stock 1 mg/mL solution of Bovine Serum Albumin (BSA), DMEM and, DMEM + 10% of BSA. MWCNTs and GFs were added separately to 3 mL of DMEM with 10% of BSA (Gibco, Cat 11020021, New Zealand) in two concentrations: 10 and 50 μg/mL of CBNs. The samples were sonicated and incubated at 37 °C, 95% RH, and 5% CO_2_ overnight. The samples were then centrifuged at 12,000 rpm for 10 min. 1 μL of the supernatant of each sample was diluted to 1:100, 1:1000 and 1:10,000 with deionized water.

### Statistical analysis

Differences between groups, ten cells per each group, were tested for statistical significance via two-way repeated measures ANOVA and Tukey multiple comparison tests using the OriginPro 8.6 software (OriginLab, Northampton, MA, USA) 100 points were measured per cell. Results were considered statistically significant at *p* < 0.05. Results are expressed as the mean ± standard deviation (SD).

### Experimental

See Fig. 8.

**Fig. 8:**
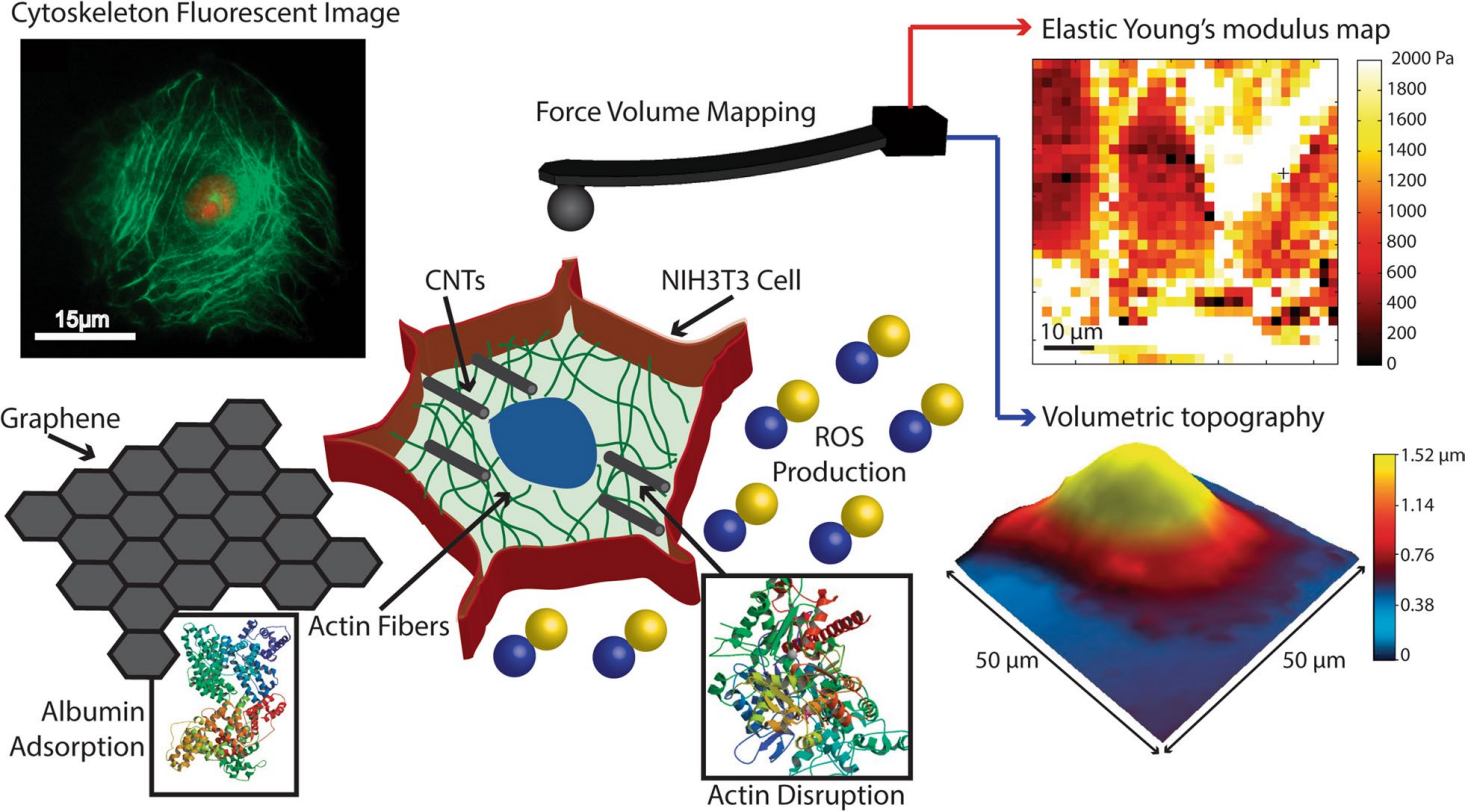
Schematic of experimental setup using AFM. The adherent cell is indented by the AFM cantilever tip the signal changes on the photodetector are transformed from voltage to distance and used to calculate the Force and using Hertz model estimating the Young modulus. The cells are exposed to CBN, the presence of these materials in the media induce ROS production, protein adsorption, and F-actin polymerization disruption

## Additional file

**Additional file 1.** TEM characterization of Multiwall Carbon Nanotubes and Graphene Oxide Flakes are showed. The LEAP images are presented for cells exposed to MWCNT and GF after 12 and 24 h measuring ROS production by DHE dye and live cells by Calcein dye.
