# Supplementary material for: Evaluation of the elastic Young’s modulus and cytotoxicity variations in fibroblasts exposed to carbon-based nanomaterials
Source: J Nanobiotechnology. 2019 Feb 23;17:32. doi: 10.1186/s12951-019-0460-8 (PMC6387485; doi:10.1186/s12951-019-0460-8)
Supplement: Supplementary file 1 — Additional file 1. TEM characterization of Multiwall Carbon Nanotubes and Graphene Oxide Flakes are showed. The LEAP images are presented for cells exposed to MWCNT and GF after 12 and 24 h measuring ROS production by DHE dye and live cells by Calcein dye. [file 12951_2019_460_MOESM1_ESM.docx]

**Additional Information**

**Evaluation of the Young’s Modulus Variations on NIH3T3 and Their Cytotoxicity Secondary to Carbon Based Nanomaterials Exposure**

TEM Images of CBNs


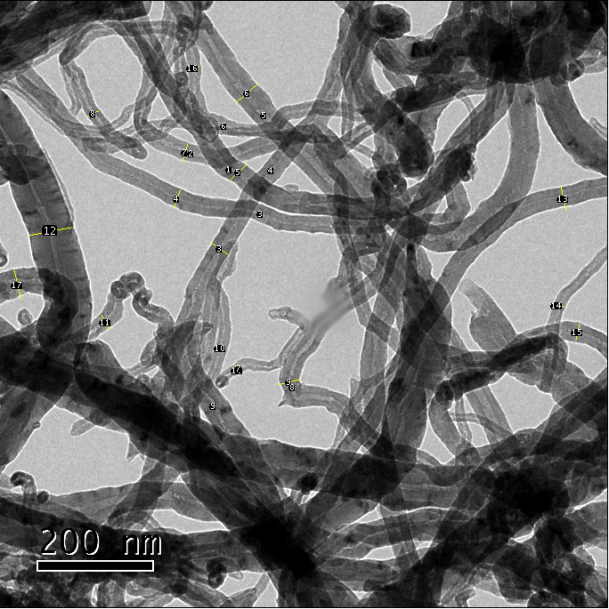




MWCNT Cat. US4311







Graphene flakes Cat. xGnP Grade H


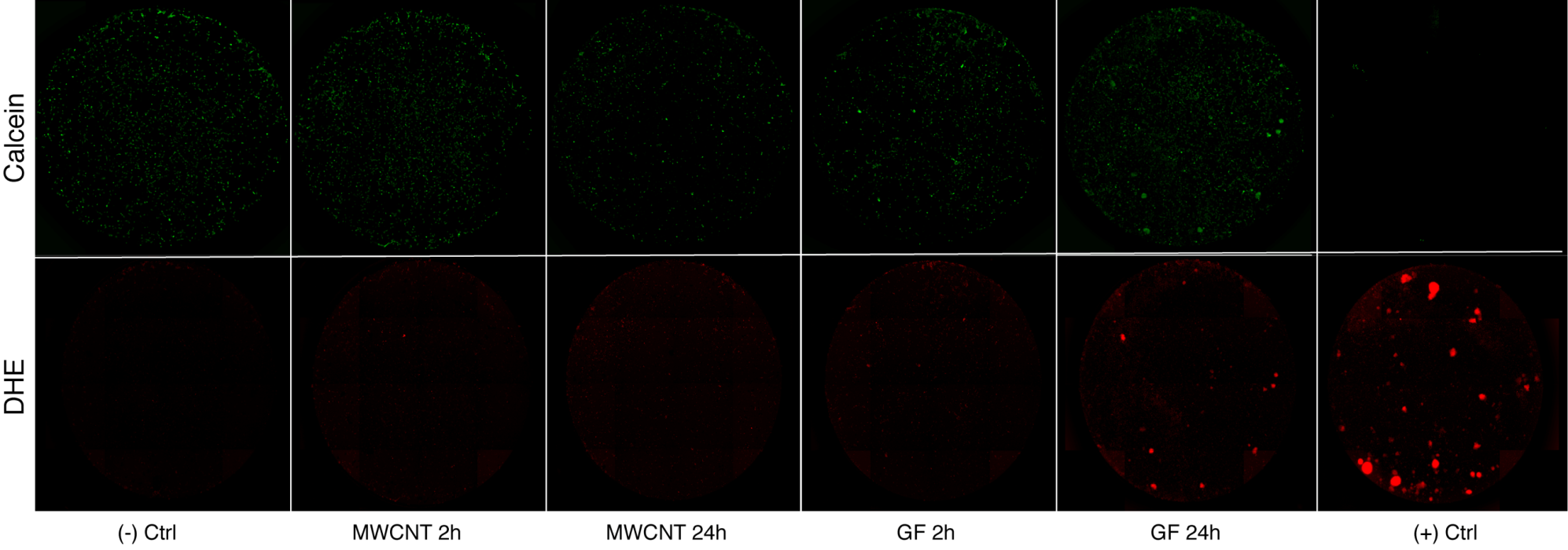


LEAP Images of Calcein and DHE fluorescence of cells exposed to MWCNT and GF at 50ug/mL
